# Digital Impressions in Implant Dentistry: A Literature Review

**DOI:** 10.3390/ijerph18031020

**Published:** 2021-01-24

**Authors:** Simone Marques, Paulo Ribeiro, Carlos Falcão, Bernardo Ferreira Lemos, Blanca Ríos-Carrasco, José Vicente Ríos-Santos, Mariano Herrero-Climent

**Affiliations:** 1 Porto Dental Institute, 4150-518 Porto, Portugal; simoneramosmarques@gmail.com (S.M.); ribeiropaulo1@gmail.com (P.R.); cfalcao@ufp.edu.pt (C.F.); blemos@ufp.edu.pt (B.F.L.); dr.herrero@herrerocliment.com (M.H.-C.); 2 Faculty of Health Sciences, Fernando Pessoa University, 4249-004 Porto, Portugal; 3 Department of Periodontology, Universidad de Sevilla, 41009 Seville, Spain; brios@us.es

**Keywords:** digital impressions, implant dentistry, intra-oral scan bodies, intra-oral scanner, accuracy, scanning technique, implant depth, implant angulation

## Abstract

Introduction. Digital impressions in implant dentistry rely on many variables, and their accuracy, particularly in complete edentulous patients, is not well understood. ***Aim.*** The purpose of this literature review was to determine which factors may influence the accuracy of digital impressions in implant dentistry. Emphasized attention was given to the design of the intra-oral scan body (ISB) and scanning techniques. Materials and methods. A Medline, PubMed and EBSCO Host databases search, complemented by a hand search, was performed in order to select relevant reports regarding the appliance of digital impressions in implant dentistry. The search subject included but was not limited to accuracy of digital impressions in implant dentistry, digital scanning techniques, the design and material of the ISBs, and the depth and angulation of the implant. The related titles and abstracts were screened, and the remaining articles that fulfilled the inclusion criteria were selected for full-text readings. Results. The literature search conducted for this review initially resulted in 108 articles, among which only 21 articles fulfilled the criteria for inclusion. Studies were evaluated according to five subjects: accuracy of digital impressions in implant dentistry; the design and material of the intra-oral scan bodies; scanning technique; the influence of implants depth/angulations on the digital impression and accuracy of different intra-oral scanner devices. Conclusions. The accuracy of digital impressions in implant dentistry depends on several aspects. The depth/angulation of the implant, the experience of the operator, the intra-oral scanner used, and environmental conditions may influence the accuracy of digital impressions in implant dentistry. However, it seems that ISBs’ design and material, as well as scanning technique, have a major impact on the trueness and precision of digital impressions in implant dentistry. Future research is suggested for the better understanding of this subject, focusing on the optimization of the ISB design and scanning protocols.

## 1. Introduction

Digital devices have had a widespread use in dental practice in the last few decades. CAD/CAM technology made it possible to fabricate implant-supported restorations through a digital workflow. Digital impressions transfer the intra-oral situation to a virtual model and represent the first step of the digital workflow. The accuracy of this procedure may determine the success of the treatment, since it is a crucial step to transfer the implant position correctly. If it is performed poorly, it can lead to a misfit of the final prosthesis, which may result at long last in mechanical and biological complications. Digital impressions can accelerate the data-capturing process and eliminate most of the drawbacks usually found with conventional impressions, thereby decreasing patient discomfort while improving the predictability of prosthesis design and manufacturing procedures [1,2].

The acquisition of a digital impression is a very user-friendly procedure that subserves the daily clinical practice. However, behind the simplicity of this procedure there is a rather complex working mechanism [3,4]. The intra-oral scanner workflow starts by emitting a light beam (laser or structured light) towards the object to be digitized. When it reaches the object’s surface, the light beam suffers a deformation, and this optic effect is captured by two or more cameras on the intra-oral scanner (IOS) devices’ tip. Then, a processing software is used to calculate the 3D coordinates (x,y,z), and creates point clouds and meshes [3,4,5]. The registration and subsequent stitching of these point clouds and meshes allows the tridimensional reconstruction of the scanned object, creating a reliable model [3,4,5,6].

When choosing an IOS device it is important not only to consider its operational features—such as the size of the intra-oral tip, the image acquisition’s speed or the ease of manipulation—but also its accuracy. Therefore, trueness and precision must be closely considered [3,4,5,6].

Trueness consists of the ability of a measurement to coincide with the real value being evaluated [5,6]. IOS’s trueness can be evaluated by superimposing a digital impression of a scanned object with a reference model of the same object, obtained by an industrial reference scanner (such as a coordinate measuring machine or an industrial optical scanner with accuracy <5 μm). Models superimposition is evaluated using reverse-engineering software in order to determine deviations mathematically [7,8].

Precision is defined as the ability of consistently taking the same measurement value. An IOS should present high trueness and precision [5,6]. IOS’s precision can be evaluated by superimposing different scans of the same object performed with the same IOS device [7,8].

Multiple in vitro studies have proven that IOSs are an important and reliable tool to capture high-quality impressions, that can be used to fabricate simple (onlays, inlays or single crowns) to complex (fixed partial prosthesis) prostheses in dentate patients [9,10,11,12,13,14].

However, due to the digital revolution in prosthodontics over the last few years, the speed of scientific papers’ publication struggles to keep up with the industry development [15,16,17,18,19], there being, to date, a very limited number of studies investigating the accuracy of digital impressions in implant dentistry [16,18]. Using this type of technology on edentulous patients is a complex procedure [16,18].

To capture the correct implant position with a digital impression it is necessary to use a specific transfer post called an intra-oral scan body (ISB) [2]. Edentulous areas can be difficult to read and mathematically interpret for IOSs, due to the lack of distinguished anatomic references, which is why having a reliable ISB design is so important to improving the accuracy of implant digital casts [16,17,18,19].

There are many factors that might compromise the performance of an IOS, when reading an implant cast, and decrease its accuracy. Regarding the equipment, aspects such as the scanning technology, the state of the device, and the temperature and illumination of the room and of the reading area may affect the accuracy of the IOS readings. The operator’s skills and experience as well as the scanning technique and sequence should also be considered as accuracy-influencing factors. In vivo, patient’s movements, limited mouth opening, and an oversized tongue may render difficult the scanning procedure [20,21,22]. In vitro, the design and material of the cast, and the design of the scan bodies as well as its light reflection properties, can affect the precision of the digital impressions. Several authors support the accuracy of this type of technology for the rehabilitation of single implants. Mangano and Veronesi conducted a randomized controlled trial comparing digital and analog workflows when restoring single implants. Both techniques showed high success rates (92%) and only 8% incidence of complications. Complications were related to biological problems (peri-implant mucositis) in patients with poor oral hygiene compliance [23]. Additionally, Ender et al. conducted a clinical trial in order to compare the precision of conventional and digital impressions in vivo, obtaining results that support the use of digital impressions in implant dentistry, comparable to those achieved for conventional techniques [21].

However, the extension of the edentulous space is one of the major obstacles when using digital workflow in implant dentistry. The lack of fixed anatomical reference points, such as teeth, leads to a superimposition of images by using the first image obtained as reference and stitching the following images to the previous ones. Each individual stitch represents a possibility for incurring an error, decreasing the accuracy of the digital impressions [23]. This misalignment error has an even higher impact when more than six implants are placed in the same dental arch [4,23,24,25,26,27,28,29]. Gimenez et al. contended that scanning larger edentulous areas significantly affected both linear and angular measurements, which can be imputed to the accumulative error of the stitching process [23]. It remains unclear in the literature from which exact number of placed implants is the decrease of accuracy clinically significant. The clinically acceptable degree of inaccuracy has been diversely discussed by many authors. Klineberg and Murray considered discrepancies of up to 30 μm at the implant–abutment interface as acceptable, and Jemt proposed a limit of 150 μm to prevent long-term complications. The misalignment error increases with the distance scanned, and consequently full arches represent a bigger challenge for IOSs [30,31]. The implant depth should also be considered because it is directly related to the scan body visibility, which can influence accuracy measurements. When the scan body is fully visible, determining the implant position is less prone to errors, meaning the deeper the implant is placed, the longer the scan body should be [23,32,33]. The literature is not in agreement on the potential influence of implant angulation [23,32,33], although most recent evidence reveals that the angulated position of the implants does not decrease the accuracy of implant digital casts [23].

As such, many factors may have an effect on the outcome and accuracy of digital impressions; further development of the scanning devices, scanning protocols, and imaging techniques is necessary to enhance the precision of the optical acquisition of implant scan bodies. Additionally, the scan body design requires further improvement in order to enhance the accuracy of digital impressions.

**Figure 1:**
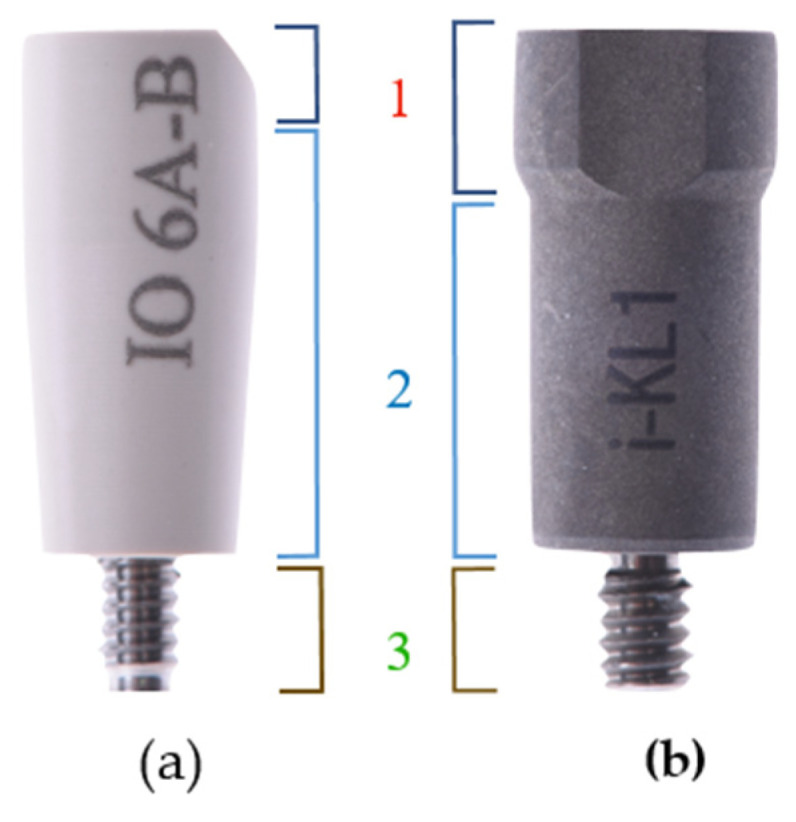
ISBs (intra-oral scan body ) have three regions: 1—scan region, 2—body and 3—base. (**a**) ISB Elos Accurate® Scan Body (Elos Medtech AB, Gothenburg, Sweden), made of PEEK. (**b**) ISB Klockner KL-1 (Klockner Implant System, SOADCO, Andorra), made of titanium.

As digital technology becomes empowered in implant dentistry, many commercial brands developed ISBs with different designs and geometries. Generally, ISBs are composed of three distinct areas: the scan region (corresponding to the upper portion), the body (corresponding to the middle portion) and the base (corresponding to the most apical portion that connects to the implant) (Figure 1). A deeply tapered connection or mismatch in materials between the base and the implant may influence the displacement of the ISB when tightened into place [34].

The scan region contains one or more scanning areas, which may have different shapes, in order to improve the accuracy of the digital impression. By incorporating an asymmetrical shape on the scan region, the surface recognition by the CAD software becomes more simple.

The majority of ISBs commercially available are made of one of two different materials, polyetheretherketone (PEEK) and titanium (Figure 1), but the body of the scan body may also contain aluminum alloy and various resins. It is important to consider the machinability of these materials and the manufacturing tolerances to improve the accuracy of ISBs. The height of commercially available ISBs ranges from 3 to 17 mm [34].

Usually, dull, smooth and opaque surfaces are easier to capture in a digital intra-oral impression than shiny, rough or translucent ones. Intraorally, it becomes very challenging due to the surface’s reflection created by saliva. Recent studies have indicated that deep, undercut, steep, sharp, angled, or crowded surfaces are also more difficult to scan, leading to less accurate point clouds. Gimenez et al. concluded that gingivally placed implants presented less scan deviation than subgingivally placed implants, regardless of the angle deviation (*p =* 0.757) [23]. It may be necessary and advantageous to create ISBs with specific characteristics for intra-oral situations. A narrow scan body, for example, may be more effective in situations with limited interproximal space, and a shorter scan body may be easier to capture in patients with complete edentulism or limited mouth opening [34].

However, in cases of edentulous jaws rehabilitation, the challenge of obtaining an accurate digital impression remains. It is necessary to create an ISB design that can be easily identified by the IOS, is accessible to manipulate by the operator, and is comfortable for the patient.

Therefore, the aim of this literature review consists of understanding the state of the art of digital impressions on implant dentistry, and understating which factors may contribute to decreasing or enhancing its accuracy, in order to attempt to provide the dental clinician with evidence-based guidelines when resorting to these impression techniques.

## 2. Materials and Methods

A MedLine, PubMed and EBSCO Host databases search was performed by two calibrated investigators (S.M. and M.H.-C.) in order to select relevant reports regarding the appliance of digital impressions in implant dentistry, using the (MeSH) keywords relevant for the main question. The guiding question of this review is “Which factors may influence the accuracy of digital impressions on implant dentistry?” Attending to the scope of influencing factors on the main question it was not possible to formulate a PICO strategy. Since the subject of digital impressions in implant dentistry is not a very old one in the literature, no time frame was applied, analyzing all studies published until May 2020. The literature search was limited to articles published in the English language. The analysis was performed according to the guidelines and references of an integrative review. Additionally, a hand search of four journals was conducted: Journal of Prostethic Dentistry (2014–present), Journal of Prosthodontics (2016–present), Clinical Implant Dentistry and Related Research (2016–present) and Clinical Oral Implants Research (2015–present).

The database search included but was not limited to the accuracy of digital impressions in implant dentistry, digital scanning techniques, the design and material of the ISBs and the depth and angulation of the implants. The employed search terms were as follows: (implant digital impressions) AND (accuracy) AND (intra oral scan body) AND (scan body design) AND ((digital scanning technique) OR (digital scanning protocol)) AND (implant depth) AND (implant angulation) AND (intra oral scanner)). However, no studies evaluating all of these features in relation to digital impressions in implant dentistry were identified.

Therefore, the database search was expanded in order to include any articles regarding digital impressions in implant dentistry, with fixed partial dentures (FPD) or full-arch prosthesis, modifying the search terms and strategy. The used search terms were then as follows: (implant digital impressions) AND (accuracy) OR (intra oral scan body) OR (scan body design) OR ((digital scanning technique) OR (digital scanning protocol)) OR (implant depth) OR (implant angulation) OR (intra oral scanner)).

Duplicated results from different databases were not considered.

Inclusion criteria comprised studies at all levels of evidence, excluding expert opinion, such as experimental clinical studies, in vitro and in vivo studies. All articles evaluating at least one of the following subjects were included: digital impressions in implant dentistry, digital scanning techniques, design and/or material of the ISBs, depth and/or angulation of the implants and performance of different IOS devices.

Exclusion criteria comprised multiple publications based on the same population and with wrong study designs. Experimental clinical studies, and in vitro and in vivo studies, that analyzed the accuracy of digital impressions only on teeth, not considering impressions on implants, were excluded.

**Figure 2:**
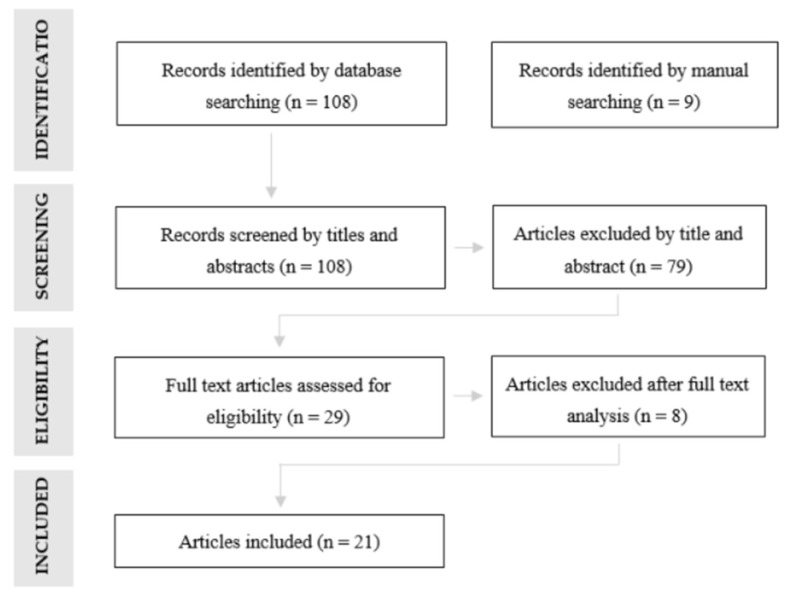
Flow chart presenting the screening of articles related to digital impressions in implant dentistry to be included in this review.

Out of 108 results, the articles were initially analyzed considering their title and abstract, excluding 79 articles because implant impressions were not considered. A full-text analysis of the 29 remaining articles were performed, excluding 8 articles, due to a lack of information regarding the obtaining method of the reference models (*n* = 3), the conventional impression materials used (*n* = 4) and the digital files superimposition technique (*n* = 1). At last, 21 articles were selected for this review (Figure 2). From a journals manual search, 9 articles were selected (2 from Journal of Prosthetic Dentistry, 2 from Journal of Prosthodontics and 5 from Clinical Oral Implants Research). However, they were all duplicated from the database search and consequently not considered. All studies from the above-mentioned search scheme were analyzed by two calibrated reviewers (S.M., M.H.C.), and screened with the inclusion/exclusion criteria.

It was not possible to perform the statistical analysis due to the report variability and the limited number of identified studies.

- From each study, the following data were extracted:
- Study design—randomized/nonrandomized controlled study, experimental study;
- Study setting—in vivo/in vitro;
- Type of impressions—digital/conventional;
- Type of arch—single-unit case, partially edentulous, completely edentulous;
- Type and number of implants placed;
- Implant depth and angulation;
- Type and design of the ISBs;
- IOS used;
- Scanning technique;
- Outcomes.

## 3. Results

A total of 21 articles were reviewed in the present study: 18 in vitro studies, 1 randomized in vitro study and 2 comparative clinical studies (Table 1 and Table 2). In total, 20 articles evaluated the accuracy of digital impressions in implant dentistry using ISBs; 5 considered the accuracy of digital impressions regarding the design and material of the ISBs; 6 focused on the accuracy of the scanning technique; 6 compared the accuracy of different IOS devices; and 8 assessed the accuracy of digital impressions concerning the depth/angulation of the implant (Table 3).

In total, 13 studies refer to completely edentulous arches with two implants (1 study), three implants (1 study), four implants (3 studies), five implants (3 studies) and six implants (5 studies). Six studies examined partially edentulous arches with one implant (one study), two implants (two studies), three implants (two studies) and with two and five implants, respectively (one study). One study evaluated partially and completely edentulous arches, with three and six implants, respectively. One study examined completely edentulous arches with no implants placed, in order to determine which IOS device and scanning strategy presented higher accuracy.

All studies reviewed are summarized in Table 4, Table 5 and Table 6.

### 3.1. Accuracy of Digital Impressions in Implant Dentistry

Twenty studies examined the accuracy of digital impressions in implant dentistry. The digital impressions accuracy outcome was evaluated by measuring linear and angular deviations or tridimensional surface deviations between reference models and test models, or by examining the fit of frameworks on test models that were fabricated on master models.

To assess linear and angular distances between implants, master models and test models were measured with coordinate measuring machines (CMM) [23,35,41,43,49]. Virtual measurements of implant distances and angulations were calculated after performing optical impressions with multiple high-precision reference scanners, such as IScan D101 (Imetric 3D Gmbh, Courgenay, Switzerland) [36], IScan D103i (Imetric, Courgenay, Switzerland) [23], IScan D104i, (Imetric, Courgenay, Switzerland) [36,42], Lava Scan ST (3M ESPE, Seefeld, Germany) [8,44], D250 (3Shape, Copenhagen, Denmark) [30,38], D800 (3Shape, Copenhagen, Denmark) [40], E3 scanner (3Shape Copenhagen, Denmark) [43], Activity 880 (Smart Optics, Germany) [45], ScanRider (Italy) [24], ATOS Compact Scan 5M (GOM GmbH, Germany) [39], COMET L3D (Carl Zeiss Optotechnik GmbH) [46] and ATOS So4 II (GOM GmbH, Germany) [47]. The STL digital values were loaded into reverse-engineering software such as Rapidform (Rapidform, INUS Technology Inc, Seoul, Korea) [19,30,35,38,40], Geomagic Qualify 12.0 (Geomagic, Morrisville, NC, USA) [8,24,31,36,42,43,44,45], Mimics **(**Materialise, Leuven, Belgium) [23], Rhinoceros 5.0 (Robert McNeel & Associates, Seattle, WA, USA) [41], Gom Inspect Professional (GOM GmbH, Germany) [39] and ATOS Professional Software (V7.5 SR2, GOM GmbH, Braunschweig, Germany) [48], and were superimposed with their respective STL master models in order to evaluate tridimensional deviations.

Attending to accuracy, it was concluded that it is viable to use a three-dimensional acquisition technology as an alternative to conventional impression procedures [23,35,42]. Giménez-González et al. registered mean linear and angular deviations for the TrueDefinition IOS from CMM measurements, from 5.38 ± 12.61 μm to –26.97 ± 50.56 μm and from 0.16° ± 0.04° to –0.43° ± 0.1°, respectively [23]. Vandweghe et al. evaluated the accuracy of four different IOS when applied for implant impressions in edentulous jaws, and concluded that the mean trueness was 0.112 ± 0,025 mm for Lava COS, 0.035 ± 0.012 mm for 3M TrueDefinition, 0.028 ± 0.007 mm for Trios and 0.061 ± 0.023 mm for Cerec Omnicam. The mean precision was 0.066 ± 0.025 mm for Lava COS, 0.030 ± 0.011 mm for 3M TrueDefinition, 0.033 ± 0.012 mm for Trios and 0.059 ± 0.024 mm for Cerec Omnicam [42]. Ciocca et al. revealed that the mean 3D position error of the digital impression was 0.041 ± 0.023 mm to 0.082 ± 0.030 mm, which is in agreement with former studies and indicates a clinically acceptable level of accuracy [43]. Menini et al. went further and compared the accuracy of conventional impression techniques with digital impressions on multiple implants, by analyzing the passive fit of a full-arch implant-supported prostheses. The Sheffield test revealed a mean gap of 0.022 ± 0.023 mm for the conventional impression and 0.015 ± 0.011 mm for the digital impression, suggesting a better accuracy of digital impressions compared to conventional ones [41]. In fact, Ribeiro et al. also concluded that for a model with four axial implants, the deviations in the digital impressions were smaller than those related to the conventional techniques [44]. However, scanning accuracy has shown to be decreased when digitizing a fully edentulous patient, compared to scanning an area of more limited extent [25]. Adriessen et al. concluded that based on the intra-oral scans obtained, the distance and angulation errors were too relevant to the manufacturers’ well-fitting frameworks for implants in edentulous mandibles. Out of 21 intra-oral scans, 5 scans presented an intra-implant distance error higher than 100 μm, 3 scans demonstrated intra-implant angulation error higher than 0.4°, with only 1 scan performing both intra-implant angulation and intra-implant distance error acceptably (lower than 0.4° and 100 μm, respectively). The lack of anatomic references for scanning on edentulous jaws appears to be the main reason for the unreliable scans [8].

### 3.2. Design/Material of ISBs

Out of the 21 studies reviewed, only 19 used ISBs on their protocol.

Despite 16 studies mentioning the shape and dimensions of the ISBs, most studies did not examined the influence of the design of the ISB on the accuracy of digital impressions [19,23,25,31,32,36,39,40,41,42,43,45,46,47,48,49]. Only four studies focused on this subject [40,47,48,49].

Three studies used prototypes [23,35,46], while 13 studies used commercially available ISBs.

All authors used screw-retained ISBs that would correspond to impression copings in conventional impressions.

Regarding dimensions, the ISBs examined had a height range between 8 and 15 mm and a diameter range varying from 4 to 5 mm.

Among all ISBs, the most commonly used design was a cylindrical shape [19,23,25,31,36,40,41,43,47,48]. Another variation of this ISB design were examined as well, having a cylindrical design with a partially beveled upper part [32,39,40,42,45,46,47,48,49].

Other designs were examined, such as flat cylinder with ball top, rectangular, cylinder with triangular region, tapered flat cylinder [47], uneven shape with bulges and indentations (cylindrical in the cervical area and slightly oval in the coronal area) and cylindrical shape with one retraction and a slightly enlarged coronal diameter [48].

All four studies that directly evaluated the influence of the ISB design on the accuracy of digital impressions agreed that the precision of scanning is dependent on the ISB surface geometry and design [31,47,48,49]. Mizumoto et al. examined five different ISB designs (Table 5). Resorting to a structured blue light industrial scanner, ISBs were scanned on an edentulous maxillary model with four dental implant analogs. Five scans of the model were performed with an IOS, applying different scanning techniques. The scans were then superimposed on the master reference model. The distance deviation and angular deviation of the ISBs was calculated. Statistical analysis was performed by using a two-factor ANOVA to analyze the influence of ISB and scanning technique on the trueness and scan time, with subsequent Tukey honestly significant difference or Bonferroni-corrected Student *t*-tests. The ISB design had a significant effect independently (*p* = 0.031). A statistically significant interaction was found between the effects of the ISB design on angular deviation (*p* <0.001) [47].

Additionally, the distance between ISBs seems to negatively impact the accuracy of digital impressions [31], and scanning time may be influenced by the ISB design [47]. Flügge et al. developed an in vitro study using two models with a different number and distribution of ISBs, produced from conventional implant impressions. These models were scanned with three different IOSs and a dental lab scanner, performing ten scans for each model and IOS. The distance and angulation between the respective ISBs were measured. The comparison of results with analysis of variance allowed us to conclude that the distance of a single tooth space and a jaw-traversing distance between ISBs revealed significantly different results for distance and angle measurements between the scanning systems (*p* < 0.05) [31]. The same author concluded with this study that the precision of ISB scanning was not significantly influenced by the detachment and repositioning of the ISB [31].

Regardless, 14 studies mentioned the material of the ISBs, but most studies did not examine its influence on the accuracy of digital impressions [19,23,25,32,35,36,39,41,42,44,46,47,48,49]. Only one study was focused on this subject [46].

The most commonly used ISB material was PEEK [19,23,25,36,38,40,46,47,48], but other materials were also examined, such as ceramics [35], metal (inox) [39], a non-specified polymer [32,39], titanium alloy [44,46], hybrids containing both titanium and PEEK [46,47,49], or PEEK and metal [47,48].

When comparing which ISB material presented a better accuracy performance, a study by Arcuri et al. concluded that PEEK was the most accurate material, followed by titanium and hybrid PEEK with titanium, respectively (Table 6) [46]. To come to this conclusion, linear and angular deviations were assessed. Considering the angular deviations, the material of the ISBs significantly influenced the expected value (*p =* 0.0232). In multivariate analysis, when the absolute values of the linear discrepancies were summed up to obtain a global measure of the linear absolute error and were considered as the response variable, a significant impact of the material of the ISBs was identified (*p* < 0.0001) [46].

### 3.3. Scanning Technique

Out of the 21 studies reviewed, only 15 studies mentioned the scanning protocol used. Four studies stated they followed the IOS manufacturer’s protocol, without specifying the procedure [31,32,42,44]. Eleven studies reported in detail the scanning technique applied [19,23,25,37,39,41,43,45,46,47,48]. However, only six studies evaluated the influence of the scanning technique on the accuracy of digital impressions. All authors concluded that the scanning protocol may influence the performance of the IOS device, and subsequently the accuracy of the digital impression [19,23,36,37,47,48].

When comparing two scanning strategies, Motel et al. achieved a significantly higher accuracy overall (*p =* 0.031) using a one-step scanning strategy with integrated ISBs (scanning both model and ISBs placed at one single time) than with using a two-step technique, which assumed scanning the model two times: first, without ISBs, and then with the ISBs positioned on the model [48].

Mizumoto et al. found a statistically significant interaction between the effects of the scan body and technique on the angular deviation (*p* < 0.001) [47].

Studies that compared digital impressions with conventional impressions claim that the splinted impression technique is more accurate compared with the non-splinted technique in edentulous patients, when using a conventional impression protocol. Papaspyridakos et al. compared splinted and non-splinted, open-tray techniques to fabricate casts that were superimposed onto reference models by optical scanning acquisition of the xyz coordinates of the implant positions for each individual cast that was realized. The paired *t*-test and Wilcoxon’s signed ranks test were used to compare the 3D discrepancies within and between splinted and non-splinted techniques, respectively. Significant difference was found at the x- and y-axis, and the 3D parameters, between the splinted group and non-splinted groups (*p* < 0.05), but not in the vertical z-axis (*p* > 0.05). Within subject, global 3D discrepancies between splinted impressions and non-splinted impressions were significantly different (*p* < 0.05), confirmed by the clinical observation of the fitting. The splinted technique generated more accurate master casts than the non-splinted technique, but in implant dentistry, digital impressions also presented accurate results [36].

When using a digital workflow and following the IOS scan protocol, a study by Giménez-González et al. revealed that the distance and angular deviations augment throughout the arch, meaning that the first scanned quadrant will always achieve better accuracy than the second quadrant. The scanning technique should take this fact into consideration [23].

Mizumoto et al. revealed that scanning techniques with different surface modifications, such as placing glass fiduciary markers on the edentulous ridge, marking the ridge and palate with pressure-indicating paste or splinting the ISBs with floss, resulted in similar distance deviations as the scanning technique without any modifications (*p =* 0.076) [47].

### 3.4. Implants Angulation/Depth

Out of 21 studies, 11 studies analyzed axial implants; 8 studies compared angulated implants with axial implants and 2 studies did not examine implants in their protocol.

Arcuri et al. evaluated the influence of the ISB position, and consequently the implant depth/angulation, on digital impressions via scanning and edentulous maxillary models with six implants placed with different depths and angulations (Table 4). The 45 scans obtained were superimposed using a best fit algorithm to a reference model, obtained with an extraoral optical scanner. Considering the angular deviations, the position of the ISBs significantly influenced the expected value (*p* < 0.0001). In multivariate analysis, when absolute values of the linear discrepancies were summed up to obtain a global measure of the linear absolute error and considered as the response variable, a significant impact of the position of the ISBs was identified (*p* < 0.0009). Therefore, it was suggested that implants’ angulation may decrease the accuracy of digital impressions [46].

Gedrimieni et al. also claimed that the angulation between implants affects the ISBs positioning and, depending on its design, may interfere with the accuracy of digital impressions [45]. However, the majority of the studies supported the theory that the accuracy of digital impressions was not influenced by different implant angulations for completely edentulous patients [23,32,39,42,44,48].

Most studies did not evaluate the impact of the implants’ vertical positioning on the accuracy of the digital impressions. Arcuri et al. also examined the implants’ depth influence using digital impressions, positioning the implants equigingivally (3 and 6 mm subgingivally). It was concluded that implant depth did not affect the final accuracy of digital impressions [46].

However, another study by Giménez-González et al., wherein implants were placed equigingivally (2 and 4 mm subgingivally), claims that the amount of visible ISB affects the accuracy of digital impressions, and so the depth of the implant should be taken into consideration when choosing the ISB design [23].

### 3.5. Accuracy of Different IOS Devices

Several devices for intra-oral optical scanning were analyzed in the reviewed studies.

Nine different IOS devices were used, as follows: Comet VZ250 (Steinbichler Optotechnik GmbH, Neubeuern, Germany) [35], Cerec Bluecam (Sirona, Bensheim, Germany) [19,37], Cerec Omnicam (Sirona, Bensheim, Germany) [25,39,42], iTero (Cadent, San Jose, CA, USA) [8,19,31,37,38], LavaCOS (3M Espe, St. Paul, MN, USA) [19,37,42], ZFX Intrascan (Zimmer, Dachau, Germany) [37], Trios (3Shape, Copenhagen, Denmark) [22,31,38,42,45,46,47,48], TrueDefinition (3M Espe, St. Paul, MN, USA) [23,24,31,39,41,42,43,44] and CS 3600 (Carestream, Rochester, NY, USA) [25].

Six studies compared the accuracy of some of the above-mentioned IOS devices. The results of the different studies are not in consensus.

In a study by van der Meer et al., when compared with iTero and Cerec Bluecam, LavaCOS resulted in the most accurate of all three scanners tested when considering mean distance errors in completely edentulous patients [19].

Contradictorily, high deviations for trueness and precision for Lava COS (*p =* 0.169) were demonstrated by a study of Vandweghe et al., which compared this IOS device with Trios (*p* < 0.001) and True Definition (*p* < 0.001). These two IOS demonstrated an acceptable accuracy for large-span implant-supported reconstructions [42]. Cerec Omnicam was less accurate compared to True Definition (*p* < 0.001) and Trios (*p* < 0.001), but no difference was found with Lava COS (*p =* 0.169). The mean trueness was 0.112 ± 0.025 mm for Lava COS, 0.035 ± 0.012 mm for True Definition, 0.028 ± 0.007 mm for Trios and 0.061 ± 0.023 mm for Cerec Omnicam. The mean precision was 0.066 ± 0.025 mm for Lava COS, 0.030 ± 0.011 mm for True Definition, 0.033 ± 0.012 mm for Trios and 0.059 ± 0.024 mm for Cerec Omnicam [42].

In a study by Imburgia et al., CS3600 and Trios performed better, showing improved accuracy results in a comparative study with four different IOS devices (CS3600, Trios, Cerec Omnicam and True Definition), on partially edentulous and fully edentulous models. Considering the same type of model used in the previous study (fully edentulous model), the mean trueness was 106.4 ± 23.1 μm for True Definition, 67.2 ± 6.9 μm for Trios, and 66.4 ± 3.9 μm for Cerec Omnicam. CS 3600 had the best mean trueness (60.6 ± 11.7 μm). In the fully edentulous model, the mean precision was 75.3 ± 43.8 μm for True Definition, 31.5 ± 9.8 μm for Trios, 57.2 ± 9.1 μm for Cerec Omnicam and 65.5 ± 16.7 μm for CS 3600 [25]. Amin et al. concluded that True Definition had significantly less 3D deviations when compared with the Cerec Omnicam [39].

Flügge et al. supported the higher precision of the IOS devices Trios and True Definition in comparison with iTero [30]. Amin et al. concluded that True Definition had significantly less 3D deviations when compared with the Cerec Omnicam [39].

When scanning edentulous jaw models with no implants placed, Patzelt et al. did not find statistically significant differences between Cerec Bluecam, LavaCOS, iTero and ZFX Intrascan, and the use of four IOSs was feasible. Nevertheless, this study concluded that IOS devices need some improvement before the recommendation use of these scanners for the digitization of edentulous jaws without any references, such as implants, in vivo [37].

## 4. Discussion

This literature review advocated to analyze the state of the art of the accuracy of digital impressions in implant dentistry, attending to the multiple variables that may have an impact on it, such as the geometry and material of ISBs, the scanning protocol, the implants depth and/or angulation, and the IOS device used.

It is important to highlight that limited high-quality evidence is available on this matter, and the interpretation of the results of this review should take into consideration the study settings presented. Therefore, the limitations of this review consist mostly of the inconsistency of study designs and protocols among the selected articles, not allowing a direct comparison of the obtained results. The risk of bias of the reviewed studies was also not assessed, which can be considered as another limitation of this literature review. However, within the mentioned limitations, the present review summarizes the state of the art of digital impressions in implant dentistry, providing the clinician some practical information concerning the factors that may influence the accuracy of this digital workflow.

In digital dentistry, the frequency of scientific publications and evidence-based articles is significantly lower than the frequency of IOS’s hardware and software updating, which creates a temporal gap and hinders the establishment of good practice guidelines.

The current evidence available on the accuracy of digital impressions in implant dentistry is mostly presented in experimental studies. Consequently, this review was mainly based on experimental studies with a low scientific evidence level. As the majority of studies on digital impressions in implant dentistry are in vitro, it is important for the clinician to carefully analyze their informative value. Only two of the reviewed studies examined the accuracy of digital impressions in vivo [8,45]. However, in vivo studies rarely present the numerical values for the accuracy of implant impressions, which excludes the possibility of a direct comparison with the outcomes of in vitro studies, since in these ones the accuracy is reported with value measurements of the deviation between reference models and test models. Further studies in vivo executed with reliable methods for outcome reporting are required.

Regardless of the study setting, the comparison of outcomes resulting from conventional and digital impressions in implant dentistry suggests that digital implant impressions are as accurate as conventional implant impressions, for fixed partial prosthesis. In full-arch rehabilitation cases, the current literature does not yet provide high-quality evidence to support the selection of implant digital impression protocols over conventional techniques. However, it presents very accurate results from the in vitro studies [23,35,41,42,43,44].

Several authors have agreed on the viability of using a tridimensional acquisition technology as an alternative to conventional impression procedures [23,35,42]. Menini et al. declared that digital impressions can be used for the fabrication of full-arch implant-supported prostheses, providing a satisfactory passive fit [41]. On the other hand, Andriessen et al. concluded that the distance and angulation errors were too significant to fabricate fitted frameworks on implants in edentulous mandibles, probably due to the lack of landmarks for scanning [8]. There are some factors that may influence the accuracy of digital impressions and that still require further investigation. For instance, many authors support the accuracy of digital impressions for the rehabilitation of single implants; however, the extension of the edentulous space and the increased number of implants are major obstacles to a full digital workflow in implant dentistry. Giménez-González et al. reported that the higher the edentulous span scanned, the more significantly affected both linear and angular measurements become, which can be imputed to the accumulative error of the images-stitching process [23]. However, it remains unclear in the literature with which exact number of placed implants does the decrease in accuracy become clinically significant [4,25,26,27,28,29].

Multiple devices for intra-oral optical scanning were analyzed in the reviewed studies, using different types of technologies. Lava COS by 3M ESPE (Seefeld, Germany) captures data in a video sequence using the principle of active wavefront sampling with structured light projection. It requires the light powder dusting of the scanning areas, in order to minimize light reflection and locate reference points for the IOS [42]. True Definition, also by 3M ESPE (Seefeld, Germany), an upgraded version of the Lava COS, is a structured light scanner which uses a pulsating visible blue light, and also works under the principle of active wavefront sampling, generating a 3D video technology. Light powder dusting with titanium oxide powder is still required [25,42]. Cerec Omnicam by Sirona (Long Island City, NY, USA) is a structured light scanner, based on the principle of confocal microscopy and active optical triangulation. This IOS does not require the powder dusting of the scanning area and also provides color information [25,42]. The Trios 3 IOS by 3Shape (Copenhagen, Denmark) is a structured light scanner, which works under the concept of confocal microscopy and ultrafast optical scanning, capturing continuously 2D images from different positions, to create a tridimensional model. Powder dusting of the scanning area is not necessary and also provides color information [25,42]. CS 3600 by Carestream (Rochester, NY, USA) is a structured LED light scanner, that works through active speed tridimensional video. It does not require powder on the scanning surface and provides high-quality color images [25].

Regarding the accuracy of different IOSs, studies by Vandweghe et al. and Imburgia et al. were consistent with the results obtained when testing the precision of True Definition (0.030 ± 0. 011 mm and 0.075 ± 0.044 mm, respectively), Trios (0.033 ± 0.012 mm and 0.032 ± 0.010 mm, respectively) and Cerec Omnicam (0.059 ± 0.024 mm and 0.057 ± 0.009 mm, respectively). When testing precision, only Cerec Omnicam obtained consistent results in both studies (0.061 ± 0.0 2 3 mm and 0.066 ± 0.004 mm, respectively). Nevertheless, the precision results obtained for True Definition (0.035 ± 0.012 mm and 0.106 ± 0.023 mm, respectively) and Trios (0.028 ± 0.007 mm and 0.067 ± 0.007 mm for Trios, respectively) did not present a significant difference, and are both clinically acceptable. Therefore, the choice of which IOS to use may not have a significant impact on the accuracy of the digital impressions, since all the latest devices present clinically acceptable results. The scanning protocol may influence the accuracy of digital impressions, and it should be taken into consideration according to the recommendations of different IOSs [25,42].

Giménez-González et al. relate that the scanning protocol followed can affect the accuracy of digital impressions. It was concluded that the distance and angular deviations were significantly increased throughout the arch from the starting point, suggesting that in partial restorations the scanning protocol should start at the area of the restoration in order to achieve the most accurate result. Additionally, the design of the ISB should be attended because the amount of visible ISB can influence the accuracy of the digital impressions. According to Giménez-González et al., when the implants are deeply placed, longer ISBs designs should be preferred, which means that the depth of the implant can also affect the accuracy of the digital impressions [23]. Although some authors have analyzed the influence of ISB design in the accuracy of digital impressions, it was not conclusive which geometry is more accurate. It appears that having an asymmetrical shape may improve the accuracy results; however, additional studies are necessary to investigate this finding as it applies to ISBs. Additionally, the ideal ISB surface material requires further investigation.

Regarding the implant angulation, the majority of the studies supported the theory that the accuracy of digital impressions was not influenced by different implant angulations for completely edentulous patients [23,32,39,42,44,48], but it should be taken into consideration that most of these studies were developed under laboratorial ambience, with optimal environment conditions, with stabilized models and without the patient-related features that may affect the digital impression, such as saliva and head movement. Further in vivo studies are required to assess the influence of implant angulation on the accuracy of digital impressions.

Currently, there is a lack of information on these topics, and more studies are required to determine the relationship between ISBs’ features, implant depth/angulation, scanning protocol and digital impression accuracy.

## 5. Conclusions

Based on the limited evidence available for this review, and considering the limitations mentioned, some preliminary conclusions can be drawn.

1. Evidence suggests that digital impressions are an accurate procedure in implant dentistry.
2. Regardless of the IOS device used, the scanning protocol can influence the accuracy of the digital impressions.
3. Implant angulation seems to have no effect on the accuracy of the digital impressions. On the other hand, implant depth may affect the accuracy of the procedure. However, clinical guidelines cannot be drawn based on the presented data.
4. ISBs are implant position transfer devices that are commercialized in multiple shapes, geometries and materials.
5. The design and material of ISBs may influence the accuracy of digital impressions.

Clinical guidelines cannot be drawn based on the current data. Further investigations focusing on the in vivo use of digital impressions in implant dentistry are required. Clinical studies and RCTs on this matter are suggested.

## Figures and Tables

**Table 1:** Study design of the included articles.

Study Design	Number of Studies
Nonrandomized clinical trial	2
Randomized experimental study	1
Experimental study	18

**Table 2:** Study setting of the included articles.

Study Design	Number of Studies
In vivo	2
In vitro	19

**Table 3:** Study main subjects.

Author/Year	Accuracy of Digital Impressions in Implant Dentistry	Design/Material of the ISBs	Scanning Technique	Implants Angulation/Depth	Accuracy of Different IOS Devices
Del Corso et al., 2009 [35]	X				
Papaspyridakos et al., 2012 [36]	X		X		
Van der Meer et al., 2012 [19]	X		X		X
Patzelt et al., 2013 [37]			X		X
Andriessen et al., 2014 [8]	X				
Lee et al., 2015 [38]	X				
Amin et al., 2016 [39]	X			X	X
Flugge et al., 2016 [31]	X			X	X
Giménez-González et al., 2016 [23]	X		X	X	
Papaspyridakos et al., 2016 [32]	X			X	
Fluegge et al., 2017 [40]	X	X			
Imburgia et al., 2017 [25]	X				X
Menini et al., 2017 [41]	X				
Vandweghe et al., 2017 [42]	X				X
Ciocca et al., 2018 [43]	X				
Ribeiro et al., 2018 [44]	X			X	
Gedrimieni et al., 2019 [45]	X			X	
Arcuri et al., 2020 [46]	X	X		X	
Mizumoto et al., 2020 [47]	X	X	X		
Motel et al., 2020 [48]	X	X	X	X	
Revilla-León et al., 2020 [49]	X	X			

**Table 4:** Summary of the reviewed studies of digital impressions in implant dentistry.

Author/Year	Study Setting	Type of Arch	Number of Implants	Implant System	Angulation of Implants	Depth of Implants	IOS Device
Del Corso et al., 2009 [35]	In vitro	Edentulous	5	3i Implant Innovations	Not reported	Not reported	Comet VZ250 (Steinbichler Optotechnik GmbH, Germany)
Papaspyridakos et al., 2012 [36]	In vitro	Edentulous	6	Not reported	Not reported	Not reported	-
Van der Meer et al., 2012 [19]	In vitro	Partially Edentulous	3	Not reported	Not reported	Not reported	-CEREC Bluecam (Sirona, Germany) -iTero (Cadent, CA, USA)-Lava COS (3M, USA)
Patzelt et al., 2013 [37]	In vitro	Edentulous	0	-	-	-	-CEREC Bluecam (Sirona, Germany)-LavaCOS (3M, USA) -iTero (Cadent, CA, USA) -ZFX Intrascan (Zimmer, Dachau, Germany)
Andriessen et al., 2014 [8]	In vivo	Edentulous	2	Strauman Standard SLA-active	Not reported	Not reported	iTero (Cadent, CA, USA
Lee et al., 2015 [38]	In vitro	Partially Edentulous	1	Strauman BL	Not reported	Not reported	iTero (Cadent, CA, USA
Amin et al., 2016 [39]	In vitro	Edentulous	5	Strauman BL	Median 3 implants; 0° Distal left implant:10° distal Distal right implant: 15°	Not reported	-CEREC Omnicam (Sirona, Germany) -True Definition (3M, USA)
Flugge et al., 2016 [31]	In vitro	Partially Edentulous	2 and 5	BL and TL	Not reported	Not reported	-iTero (Cadent, CA, USA)-Trios (3Shape, Denmark)-True Definition (3M, USA)
Giménez-González et al., 2016 [23]	In vitro	Edentulous	6	Certain implants Biomet 3i	#2 #4 #7 #15: 0° #4 #13: 30°	#2 #4 #13 #15: 0 mm #7: 4 mm #10: 2 mm	True Definition (3M, USA)
Papaspyridakos et al., 2016 [32]	In vitro	Edentulous	5	Strauman BL	Median 3 implants: 0° Distal left implant: 10° Distal right implant:15°	Not reported	Trios (3Shape, Denmark)
Fluegge et al., 2017 [40]	In vitro	Partially Edentulous	2	S1 Camlog	Not reported	Not reported	-
Imburgia et al., 2017 [25]	In vitro	Edentulous and Partially Edentulous	3 and 6	BT Safe Int ^®^, BTK- Biotec Implants	0°	Not reported	-CS3600 (Carestream, USA)-Trios3 (3Shape, Denmark) -CEREC Omnicam (Sirona Germany) -True Definition (3M, USA)
Menini et al., 2017 [41]	In vitro	Edentulous	4	Biomet 3i	Not reported	Not reported	True Definition (3M, USA)
Vandweghe et al., 2017 [42]	In vitro	Edentulous	6	IBT, Southern Implants	#46-44: 0,57° ° #44-42: 1,65° #42-32: 4,62° #32-34: 4,79 #34-36: 4,22°	Not reported	-Lava COS (3M, USA) -True Definition (3M, USA) -CEREC Omnicam (Sirona, Germany) -Trios (3Shape, Denmark)
Ciocca et al., 2018 [43]	In vitro	Edentulous	6	Premium Kohno, Sweden and Martina	Not reported	Not reported	True Definition (3M, USA)
Ribeiro et al., 2018 [44]	In vitro	Edentulous	4	Klockner KL RP implants	Model 1: 0° Model 2: divergence angle of 15° between the more distal implants and convergence angle of 15° between the two central implants	Model 1: 0 mm Model 2: 0 mm	True Definition (3M, USA)
Gedrimieni et al., 2019 [45]	In vivo	Partially Edentulous	2	AnyOne Megagen	10°	Not reported	Trios (3Shape, Denmark)
Arcuri et al., 2020 [46]	In vitro	Edentulous	6	Not reported	#16: 0° #14: 25° distal #12 #22: 0° #24: 20° distal #26: 20° distal 20° facial	#16: 6 mm #14: 3 mm #12 #22 #24: 0 mm #26: 2 0 mm	Trios (3Shape, Denmark)
Mizumoto et al., 2020 [47]	In vitro	Edentulous	4	TSV Zimmer Biomet	0°	3 mm	Trios (3Shape, Denmark)
Motel et al., 2020 [48]	In vitro	Edentulous	3	Nobelreplace Select implants, Nobel Biocare	Mesial Implant: 15° vestibular Central and Distal implant: 0°	Not reported	Trios (3Shape, Denmark)
Revilla-León et al., 2020 [49]	In vitro	Partially Edentulous	3	RP Branemark system; Nobel Biocare	0°	Not reported	-

**Table 5:** Summary of the reviewed studies of digital impressions in implant dentistry (additional columns).

Author/Year	ISB Type	ISB Design	ISB Material	Reference Method	Superimposition Software
Del Corso et al., 2009 [35]	Prototypes	Reference markers were assembled on a stainless steel-type AISI-310 base referencing the implant position	Ceramic	CMM	-
Papaspyridakos et al., 2012 [36]	Not reported	Cylindrical with 5 mm diameter and 15 mm height	Metallic (inox)	Scan D101 (Imetric 3D Switzerland)	Imetric, Courgenay, Switzerland
Van der Meer et al., 2012 [19]	Createch Medical (Createch Medical, Spain)	Cylindrical	PEEK	Leitz PMM 12106	Rapidform, INUS Technology Inc, Seoul, Korea
Patzelt et al., 2013 [37]	-	-	-	Activity 101 (Smart Optics, Germany)	(Geomagic Qualify 12, 3D Systems, Rock Hill, SC, USA)
Andriessen et al., 2014 [8]	Regular Neck scan abutment (Straumann, Switzerland)	Not reported	Not reported	Lava Scan ST (3M, Germany)	(Geomagic Qualify 12, 3D Systems, Rock Hill, SC, USA)
Lee et al., 2015 [38]	Regular Neck scan abutment (Straumann, Switzerland)	Not reported	Not reported	Lava Scan ST (3M, Germany)	(Geomagic Qualify 12, 3D Systems, Rock Hill, SC, USA)
Amin et al., 2016 [39]	RC (Straumann, Switzerland)	Flat and cylindrical with a partially beveled upper part	Polymer	Activity 880 (Smart Optics, Germany)	(Geomagic Qualify 12, 3D Systems, Rock Hill, SC, USA)
Flugge et al., 2016 [31]	Not reported	Model 1: 1 TL scanbody H: 10 mm, ∅: 5 mm and BL scanbody H: 9 mm, ∅: 4 mm Model 2: 5 TL scanbody H: 10 mm, ∅: 5 mm	Not reported	D250 (3Shape, Denmark)	Rapidform, INUS Technology Inc, Seoul, Korea
Giménez-González et al., 2016 [23]	Prototypes	Cylindrical with 8 mm height	PEEK	CMM	(Geomagic Qualify 12, 3D Systems, Rock Hill, SC, USA)
Papaspyridakos et al., 2016 [32]	RC (Straumann, Switzerland)	Flat and cylindrical with a partially beveled upper part	Polymer	IScan D103i (Imetric, Switzerland	Mimics (Materialise, Belgium)
Fluegge et al., 2017 [40]	-23: (REF K2600.3810) -25: (REF K2600.4310) -35: (REF 048.168)-36: (REF 025.4915)	-23/25: Height of 10 mm and a diameter of 4.3 mm (23/25) -35: Height of 10 mm and a diameter of 5 mm -36: Height of 9 mm and a diameter of 4 mm	Not reported	D250 (3Shape, Denmark)	Rapidform (Rapidform, Korea)
Imburgia et al., 2017 [25]	BT Scanbodies^®^, BTK-Biotec Implants, Italy	Cylindrical	PEEK	ScanRider (Italy)	(Geomagic Qualify 12, 3D Systems, Rock Hill, SC, USA)
Menini et al., 2017 [41]	Createch Medical (Createch Medical, Spain)	Height of 8 mm and a diameter of 4 mm	PEEK	CMM	Rapidform (Rapidform, Korea)
Vandweghe et al., 2017 [42]	Proscan, Zonhoven, Belgium	Cylindrical shape with an axial incision	PEEK	IScan D104i (Imetric, Switzerland)	(Geomagic Qualify 12, 3D Systems, Rock Hill, SC, USA)
Ciocca et al., 2018 [43]	Sweden and Martina	8–10 mm height	Not reported	OCMM (SmartScope Flash CNC 300; Optical Gaging Products, Rochester, NY, USA)-error (MPE) < 3.5 μm	Rhinoceros (Rhinoceros 5.0; USA)
Ribeiro et al., 2018 [44]	Not reported	Not reported	Titanium	IScan D104i (Imetric, Switzerland)	(Geomagic Qualify 12, 3D Systems, Rock Hill, SC, USA)
Gedrimieni et al., 2019 [45]	3 shape	Flat and cylindrical with a partially beveled upper part	Not reported	D800 (3Shape, Denmark)	Rapidform (Rapidform, Korea)
Arcuri et al., 2020 [46]	Prototypes	Cylindrical shape with an axial Incision; 4.1mm diameter, height 9 mm; ± 0.01mm tolerance	PEEK Titanium Hybrids (PEEK body and Ti base)	ATOS Compact Scan 5M (GOM GmbH, Germany)	Parametric measurement software (Gom Inspect Professional, GOM GmbH, Germany)
Mizumoto et al., 2020 [47]	1. AF (IO-Flo; Dentsply Sirona) 2. NT (Nt-Trading GmbH & CoKG 3. DE (DESS-USA) 4. C3D (Core3Dcentres) 5. ZI (Zimmer Biomet Dental)	1. Flat cylinder with ball top. 2. Rectangular 3. Cylinder with triangular region. 4. Tapered flat cylinder 5. Flat cylinder	1. PEEK/Metal (base) 2. PEEK/Metal 3. PEEK/PEEK 4. PEEK/Ti 5. PEEK/PEEK	COMET L3D (Carl Zeiss Optotechnik GmbH)	Industrial metrology software program (Polyworks; InnovMetric Software Inc)
Motel et al., 2020 [48]	1. Elos A/S 2. NT-trading, GmbH 3.TeamZiereis, GmbH.	1. The body of the ISB presents a flat and cylindrical shape. The scan region presents a partially beveled segment. 2. The ISB presents an asymmetrical shape with bulges and indentations. The body of the ISB is cylindrical and the scan region is light. 3. The ISB presents a cylindrical shape with one retraction each and a slightly enlarged diameter in the scan region. ISB 3 presents an intermediate shape between ISB 1 and ISB 2.	1. Titanium / PEEK 2. PEEK/Metal 3. PEEK	ATOS So4 II (GOM GmbH, Germany)	ATOS Professional Software (V7.5 SR2, GOM GmbH, Germany)
Revilla-León et al., 2020 [49]	1. Elos Medtech 2. Nt-Trading 3. Dynamic Abutment	1. Cylinder with angled flat surface, one-piece screw-retained ISB geometry 2. One-piece screw-retained ISB geometry 3. Two-piece screw-retained/ magnet-retained ISB geometry	1. Titanium base, PEEK 2. Titanium base, PEEK 3. PEEK	E3 scanner (3 Shape, Denmark) and CMM	Geomagic Qualify 12.0 (Geomagic, USA)

**Table 6:** Summary of the reviewed studies of digital impressions in implant dentistry (additional columns).

Author/Year	Scanning Technique	Outcomes
Del Corso et al., 2009 [35]	Not reported	Regarding the accuracy, it appears that 3D scanning technologies are valid options for conventional impressions techniques. Nevertheless, the bias levels presented in this study need confirmation in a clinical trial.
Papaspyridakos et al., 2012 [36]	-	When comparing splinted and non-splinted impression techniques in edentulous patients, the first one shows better accuracy results. The positioning of the implant in the dental arch affected the accuracy of the impressions. Considering the implant system used (external connection), a 3D misfit ranging from 59 to 72 mm is considered as the highest discrepancy in order to obtain an acceptable clinical fit with one-piece implant fixed complete dental prosthesis.
Van der Meer et al., 2012 [19]	Attending to the scanning of implant locators, the IOS manufacturers were asked about the high-accuracy scanning protocol, as well as special recommendations or technique modifications, considering the clinical situation on the stand. iTero and CEREC had only one recommended scanning technique for all cases and did not make a distinction between normal scanning and high-accuracy scanning. Lava COS presented a high-accuracy scanning protocol and subsequent calibration protocol. When scanning implant abutments, the LAVA COS high-accuracy scanning technique consists of a calibration with a small calibration block before beginning the intraoral scan, followed by a slow zig-zag scanning of the dental arch. Then, the calibration block is once again performed. The calibration measurements are used to calculate and compensate for deviations that might occur during the scanning procedure.	Lava COS performance with a high-accuracy scanning technique obtained the lowest and most consistent errors of all 3 IOS devices when considering the mean distance errors in full arch impressions. The increased distance and/or angular errors over the length of the dental arch can be explained by the accumulation of errors of the patched tridimensional areas, but the rebounds were not statistically significant.
Patzelt et al., 2013 [37]	The scanning protocol started at the distobuccal surfaces, following the crest to the opposite surface and finally completing the palatal gaps by rolling the scanner tip in a zig-zag trajectory over the palate. On the mandibular area, the scanner tip was used in a zig-zag trajectory, initiating at the distal area of one surface and following the jaw crest to the antagonist surface.	The digitization of edentulous mandible models was feasible with the use of four IOS devices. The high levels of inaccuracy lead to the conclusion that enhancements are needed before the clinical recommendation of the use of these scanners for the digitization of edentulous jaws in vivo.
Andriessen et al., 2014 [8]	Not reported	Considering the intra-oral scans obtained of the edentulous mandibles, it was not possible to produce well-fitting frameworks on implants, because the distance and angulation errors were too significant. The lack of anatomic landmarks for scanning seem to be the reason for these unreliable results.
Lee et al., 2015 [38]	Not reported	Milled models obtained from the digital impression with IOS can be compared to gypsum models obtained from the conventional impression in most anatomical areas. However, in areas such as grooves and fossae, conventional models presented a more thorough and detailed anatomy. Vertical displacements of implant position from both groups were statistically significantly different from the reference model.
Amin et al., 2016 [39]	The scanning protocol was initiated at the right retromolar pad, performing a continuous scanning movement through the occlusal surface until the left retromolar pad. The IOS tip was placed again at the right retromolar pad, performing a continuous scanning movement through the buccal surface until the left retromolar pad. The same procedure was used to capture the lingual surface. Voids and gap areas were re-scanned in the end by using the right retromolar pad as reference.	True Definition and Omnicam provided significantly more accurate impressions than the conventional techniques, on full arch implant impressions. True Definition presented significantly less tridimensional deviations than Omnicam.
Flugge et al., 2016 [31]	Following the manufacturer’s recommendation.	The scanning precision of IOSs is significantly different among the different tested devices. The precision of the IOS systems decreased with an increasing distance between ISBs, whereas the precision of the extraoral reference scanner was not influenced by the distance between ISBs.
Giménez-González et al., 2016 [23]	The scanning protocol was performed according to the manufacturer, using the IOS camera parallel to the gum. Scanning protocol started at mesial #15, describing a circular movement around ISB #15, and kept on scanning the gingiva surrounding all the ISB (using the same circular trajectory), until digitizing the full anterior span between #10 and #7. The IOS camera was then moved back to the previous scan data near ISB #10, performing a 180° turn of the IOS tip toward the other quadrant. The same scan technique around the contralateral ISB was used. At the end, each of the ISB was captured through tilting the camera and designing a circle around the ISB body region.	The portion of visible ISB influences the final accuracy of the digital impression, so when the implants are placed more deeply, longer ISBs are recommended. The experience of the operator may influence the accuracy of the digital impression, as well as the individual ability to follow a specific scan protocol. Since the distance and angular deviations were increased throughout the arch, it is advisable to start scanning the area of the arch where the restoration is needed, in the case of partial restorations. The angulation of the implants did not influence the accuracy of the digital impressions.
Papaspyridakos et al., 2016 [32]	Following the manufacturer’s recommendation.	On edentulous patients, the accuracy of digital impressions and conventional impressions using splinted technique was similar, and both achieved better results than conventional impressions using the non-splinted technique. The accuracy of implant impressions is not influenced by the implant angulation up to 15° for completely edentulous patients.
Fluegge et al., 2017 [40]	Not reported	The precision of extraoral scanning of ISBs is influenced by the ISB surface design and geometry and by the distance between ISBs, but not by the detachment and repositioning of the ISBs.
Imburgia et al., 2017 [25]	For every IOS device, the scanning protocol comprised a zig-zag trajectory: starting from the first quadrant, the tip of the IOS draws an arch movement, from vestibular to palatal and back, slowly moving forward, passing over the occlusal surface.	Significant differences in trueness were found among different IOSs, but no significant differences in precision were found. CS3600 had the best trueness results. Nevertheless, Trios 3 had better results in the transition from the partially to the fully edentulous model. Scanning a fully edentulous jaw remains more difficult than scanning an area of more limited extent.
Menini et al., 2017 [41]	The scanning protocol started from #26 and a first overall scan was performed in a continuous movement around all the ISBs, reaching #16. Then, another scan of each ISB was accomplished, making a circular movement. Voids and gap areas were re-scanned in the end. The entire scanning process had to be concluded in less than seven minutes.	IOS is a reliable alternative to conventional impression materials for the fabrication of full-arch implant-support prosthesis, providing an acceptable passive fit.
Vandweghe et al., 2017 [42]	Following the manufacturer’s recommendation	There was a significant difference in accuracy between the different IOS devices. Lava COS demonstrated the highest deviations for trueness and precision and performed significantly worse compared to the other IOS.
Ciocca et al., 2018 [43]	During the entire scanning procedure, the camera tip was placed parallel to the gingiva, following the dental arch. The scanning protocol started from position #46, performing circular movements around each ISB towards position #36. All the gaps were improved, making a scan back to the position #46.	Operator skill and experience did not affect the accuracy of digital impressions. The mean 3D position error of the digital impression was 0.041 ± 0.023 mm to 0.082 ± 0.030 mm, suggesting an acceptable accuracy result. Regardless of the scanning technique, the distance between ISBs influenced the magnitude of the error. Errors increased with an increasing length of scan within the arch.
Ribeiro et al., 2018 [44]	Following the manufacturer’s recommendation.	The deviations found in the digital impressions group were smaller than those related to conventional impressions, when using parallel implants. The same cannot be stated for angled implants, where the results between groups were similar.
Gedrimieni et al., 2019 [45]	On the maxilla, the scanning protocol initiated on the occlusal surface, moving then towards the buccal and palatal surfaces. On the mandible, the occlusal surfaces were scanned first. Thereafter, scanning of the lingual and buccal surfaces was performed.	The angulation between implants affected the positioning of the ISBs and the accuracy of digital impressions.
Arcuri et al., 2020 [46]	Followed manufacturer recommendations. From #26 to #16, the scanning protocol had as its starting point the occlusal–palatal ISB surface, maintaining an approximate 45° inclination of the IOS tip and performing a wave movement in the anterior area, in order to avoid image splitting. The buccal area and any other gaps were re-scanned thereafter.	Considering the ISB material, the accuracy performed better as follows: PEEK > Ti > PEEK + Ti. Angulation of the implants decreases the accuracy of digital impressions. Depth of the implant seems to have no influence on the accuracy of digital impressions.
Mizumoto et al., 2020 [47]	A standardized scan path was used according to the manufacturer’s recommendation, which consisted of scanning the occlusal surface, then the buccal surface, and then the palatal surface. 1. Unmodified master model (NO). 2. Glass fiduciary markers placed on the edentulous ridge (GB). 3. Pressure-indicating paste brushed over the ridge and palate (PP). 4. Floss tied between the scan bodies (FL).	The accuracy of full-arch digital implant impressions using ISBs was affected by both the ISB and scan protocol when using one specific IOS system. The ZI scan body had significantly less distance deviation, whereas splinting ISBs with floss led to significantly more distance deviation. Scan techniques with different surface modifications resulted in similar distance deviations to the technique without any modifications. The use of different ISBs led to significant differences in scan time.
Motel et al., 2020 [48]	Strategy A was a one-step procedure that included both the titanium master model and the integrated scan bodies. Strategy B comprised two steps. First, a digital overlay was performed with a scan of the titanium master model without integrated scan bodies. A second scan was performed with the titanium master model and integrated scan bodies.	The quality of digital intraoral impressions seems to be influenced by both the geometry of the scan body and the scan strategy. For clinical practice, the one-step scan strategy seems beneficial. The high accuracy in the use of 3Shape’s ISB leads to a corresponding clinical recommendation.
Revilla-León et al., 2020 [49]	-	The lower overall tridimensional discrepancy was registered in the Elos Medtech < Dynamic Abutment < NtTrading < CNV. The 3 scan bodies and digital implant replica systems evaluated obtained better accuracy in the 3D implant position transference than conventional procedures.

## Data Availability

Not applicable.
